# Maternal mental health and economic autonomy in lowland rural Nepal: Do parents-in-law provide constraint or support?

**DOI:** 10.1093/emph/eoad020

**Published:** 2023-07-04

**Authors:** Akanksha A Marphatia, Laura K Busert-Sebela, Lu Gram, Mario Cortina-Borja, Alice M Reid, Dharma S Manandhar, Jonathan C K Wells, Naomi M Saville

**Affiliations:** Department of Geography, University of Cambridge, Cambridge, UK; Population, Policy and Practice Research and Teaching Department, Great Ormond Street Institute of Child Health, University College London, London, UK; Population, Policy and Practice Research and Teaching Department, Great Ormond Street Institute of Child Health, University College London, London, UK; Institute for Global Health, University College London, London, UK; Population, Policy and Practice Research and Teaching Department, Great Ormond Street Institute of Child Health, University College London, London, UK; Department of Geography, University of Cambridge, Cambridge, UK; Mother and Infant Research Activities, Kathmandu, Nepal; Population, Policy and Practice Research and Teaching Department, Great Ormond Street Institute of Child Health, University College London, London, UK; Institute for Global Health, University College London, London, UK

**Keywords:** maternal mental health, autonomy, parents-in-law, kin dynamics, household residence, rural Nepal

## Abstract

**Background and objectives:**

In patrilocal societies, married women typically co-reside with their parents-in-law, who may act in their son’s reproductive interests. These relationships may shape maternal mental health and autonomy. Few studies have examined these dynamics from an evolutionary perspective. Theoretically, marital kin may increase their fitness by increasing maternal investment or by reducing paternity uncertainty. We explored how co-residence with parents-in-law and husband is associated with maternal outcomes to evaluate whether marital kin provide support or constraint.

**Methodology:**

We analysed data from 444 households in rural lowland Nepal. Maternal mental health was assessed by General Health Questionnaire. Logistic regression models investigated whether, relative to mothers living with both husband and parents-in-law, those co-resident with other combinations of relatives had poorer mental health and lower household economic autonomy (decision-making, bargaining power), adjusting for socio-economic confounders.

**Results:**

Co-residence with husband only, or neither husband nor parents-in-law, was associated with higher odds of mothers reporting feeling worthless and losing sleep but also earning income and making household expenditure decisions. Husband co-residence was associated with overall maternal distress but also with less unpaid care work and greater decision-making responsibility. There were no differences in maternal outcomes for mothers living with parents-in-law only, relative to those living with both husbands and parents-in-law.

**Conclusions and implications:**

Co-residence of parents-in-law and husbands was associated with contrasting patterns of maternal mental health and economic autonomy. We suggest that different marital kin place different economic demands on mothers, while restricting their autonomy in different ways as forms of ‘mate-guarding’.

## BACKGROUND

Social cooperation is a hallmark of human evolution [1], closely associated with the expansion of the brain in the genus *Homo*. However, at an empirical level, evolutionary perspectives on cooperation have been dominated by research on men, with most studies in high-income western societies [2]. Addressing the experience of cooperation among women in non-western settings, and its health impacts, is therefore a priority [2]. Our study addresses this issue in rural lowland Nepal, focusing on the mothers of children enrolled in a birth cohort.

When it comes to the social component of being a mother, evolutionary biologists have offered two overarching frameworks for our species that have very different implications for social interactions. First, humans are widely presented as cooperative breeders, where costs and responsibilities of raising children are shared by mothers with various other members of the family and social community [3, 4]. Notably, these ‘others’ are not necessarily the mother’s husband/partner and are often other women such as relatives, neighbours or friends [3, 5]. Second, the value of cooperation is predicted to increase with genetic relatedness, promoting shared interests in childcare [6, 7]. On this basis, reduced cooperation, or even tension, might be expected between individuals that are not genetically related.

From a genetic perspective, the ideal source of social support for a mother is expected to be her own mother (maternal grandmother), with whom 50% of alleles are shared. No other category of relative is guaranteed to share such a high proportion of alleles with the mother. Among many non-human primates, as well as most mammals, most species are philopatric and females do indeed remain close to their natal kin [8]. Humans are a notable exception to this pattern, however, as adult women may live with either their maternal kin or their husband’s kin, or in nuclear households, depending on the society and circumstances. This brings into focus the extent to which individuals living in the same household are genetically unrelated. In patrilineal patrilocal societies, for example, mothers commonly reside with their parents-in-law [9], whereas in matrilineal matrilocal societies, they typically reside with their own parents [10].

To date, evolutionary studies of household composition have focused primarily on the implications of the presence/absence of different relatives for maternal fertility and child health. In low-income settings with high mortality risk, for example, the presence of maternal grandmothers has been associated with greater support for maternal reproduction [10, 11], providing advice on child care [12], and better survival and nutritional status of grandchildren [13–15], though some studies are not consistent [6]. In this way, maternal grandmothers may increase their own fitness through maintaining supportive and cooperative relationships with their daughters [16]. Conversely, the mother’s parents-in-law have often been presented in negative light, due to the expectation that they will act in the fitness interests of their son and grandchildren, potentially at a cost to the mother’s own interests [17]. In traditional South Asian societies, for example, where married women generally and live in extended family households, parents-in-law may constrain mothers’ agency and access to resources [18, 19], though again some studies show supportive associations [16, 20–22]. Overall, lack of support and psychosocial stress may adversely affect mothers’ ability to care for their children [23–25], and hence impact maternal fitness.

Recently, the evolutionary theoretical framing of parents-in-law was challenged by Dyble *et al*. [26], on the grounds that despite only being relatives through marriage, parents-in-law can still gain indirect fitness benefits through cooperating with and supporting their son’s wife. For example, mothers draw on different forms of embodied and social ‘maternal capital’ to invest in their offspring [27]. If genetically unrelated family members were to promote maternal health and autonomy as forms of maternal capital, they could gain fitness rewards, through promoting maternal investment in offspring with whom they share alleles. What, therefore, might constrain the support of parents-in-law for mothers?

**Figure 1.**
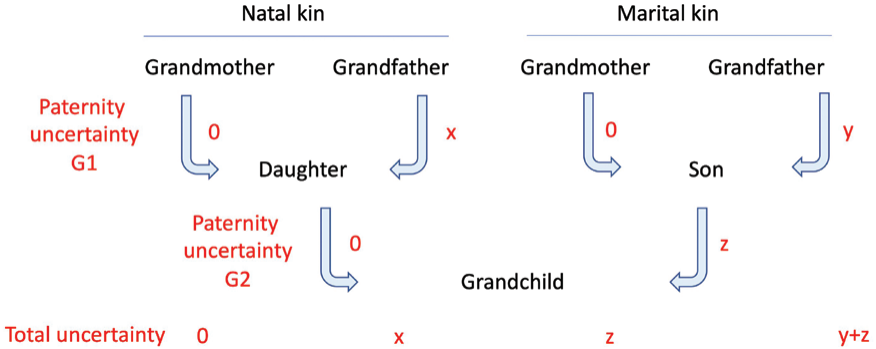
Schematic diagram illustrating the impact of potential paternity uncertainty across two generations (G1, G2) on the genetic association of maternal and paternal grandparents with grandchildren. The terms x, y and z (or zero) indicate the probability of paternity uncertainty for each grandparent in abstract units. Paternity uncertainty in G1 has the same implication for all grandchildren, whereas paternity uncertainty in G2 may vary across each grandchild

A fundamental difference for maternal versus husband’s kin concerns paternity uncertainty. When this is zero in both generations, all four grandparents will share 25% of alleles with each grandchild. If paternity uncertainty is >0, however, each grandparent has a different pathway through which the likelihood of being genetically related to grandchildren is determined (Fig. 1). Only the maternal grandmother remains guaranteed to share 25% of her alleles with each grandchild. Consequently, the husband’s relatives could promote their own fitness not by supporting the mother but simply by controlling her behaviour (often termed ‘mate-guarding’ [28, 29]) in order to minimize paternity uncertainty. Moreover, with no direct ‘genetic stake’ in their son’s wife, parents-in-law could demand from the mother levels of investment in grandchildren and unpaid care work that impose substantial costs on the mother herself. For example, high levels of fertility (benefitting the fitness of parents-in-law) may lead to chronic maternal undernutrition, a scenario recognized in public health as ‘maternal depletion’ syndrome [30, 31]. If this strategy were to severely impair the mother’s economic or childcare productivity, parents-in-law could potentially minimize any fitness costs by replacing her with another wife for their son, while keeping the first wife’s offspring. There may even be an economic incentive for this, where wives bring dowry with marriage.

Paternity uncertainty takes on particular importance in patriarchal patrilocal societies, common in South Asia, where the transfer of material property down the male line (representing long-term paternal investment) will only benefit the alleles of a woman’s marital kin if her offspring are indeed her husband’s [32]. This perspective may help understand why norms for controlling the behaviour of women of reproductive age are common in such societies. In the Maithili-speaking Madhesi population of lowland Nepal where our study was carried out, for example, mothers-in-law and their husbands act as ‘household guardians’ and maintain a strong hierarchy over younger household members including their sons. It is not culturally appropriate for daughters-in-law, particularly when newly married, to go outside the house except for specific reasons such as accessing healthcare, when they would be accompanied by the mother-in-law or husband [19]. O’Hara and Clement [33] described a set of entrenched social norms in Nepal that collectively maintain women’s subordinate position: that women should obey their husbands in all things; that women’s work should be limited to household chores; that education is not valuable for women; that women should leave politics to men; that there are times when women deserve to be beaten; and that a woman should tolerate violence in order to keep her family together. While individuals vary in the extent to which they hold these norms [33], the prevalence of violence against women is common in Nepal [34], especially in the lowland Terai region [35], and the fear of violence is likely to be one factor that acts to maintain women’s subservience. The curtailment of women’s independence can be seen as a form of exploitation of the daughter-in-law by members of her household that are genetically unrelated to her, while also minimizing the likelihood of paternity uncertainty.

From a different theoretical perspective, the role of the parents-in-law in family dynamics has been approached through the lens of the ‘patriarchal bargain’ [36]. According to this perspective, a young bride is obliged to accept a position subordinate to both men and senior women in the marital household. The daughter-in-law may serve the mother-in-law’s own interests, including her wellbeing, health and food security in old age. In the short term, the daughter-in-law’s labour and reproduction are subordinate to, and dependent on, the economic interests of male household members, with whom the mother-in-law is aligned, but in the long-term, the daughter-in-law may gain her own authority, eventually at the expense of her own daughter-in-law when her son marries [36]. Like evolutionary approaches, this framework predicts conflict between household members, whereby parents-in-law favour a subservient daughter-in-law who will not challenge their influence over their son.

Based on the evolutionary framework set out above, we therefore predict that the mental health of a woman living with her husband’s kin may be strongly shaped by whether household relations are more supportive, or more controlling and exploitative. We further predict that her mental health may depend on which relatives are co-resident. For example, the behaviour of parents-in-law might change if the husband has temporarily migrated away for work, and can neither contribute directly to childcare nor guarantee his wife’s fidelity.

To date, studies addressing the mental health of mothers in their own right do not tend to use an evolutionary perspective [37]. Nevertheless, research from South Asian countries indicates that mothers with difficult relationships with their parents-in-law may experience psychological distress [38–40], especially during the antenatal and postnatal periods [41–43]. In Nepal, studies have shown that women who were pressured into childbearing by their mothers-in-law, particularly to have a son, and those with a greater number of children, tend to be more distressed [44, 45]. For Maithili-speaking Madhesi women in Nepal with limited autonomy in household decision-making, psychological distress is also related to the pressure to be a dutiful wife and mother [38]. In Nepal and Pakistan, women living in extended families may also be exposed to intimate partner violence, which negatively affects their mental health [46–48].

### Study aim and hypotheses

Our study aims to shed new light on the extent to which mothers are constrained or supported by members of the household to whom they are genetically unrelated, and to identify which members are more influential for maternal mental health and economic autonomy. We address mental health through the lens of psychological distress, using markers of depression and anxiety [49]. Economic autonomy relates to a mother’s ability to influence the allocation of household resources in line with her own goals and desires [50]. Our measures of economic autonomy incorporate both decision-making power and bargaining power over household economic activities.

**Figure 2.**
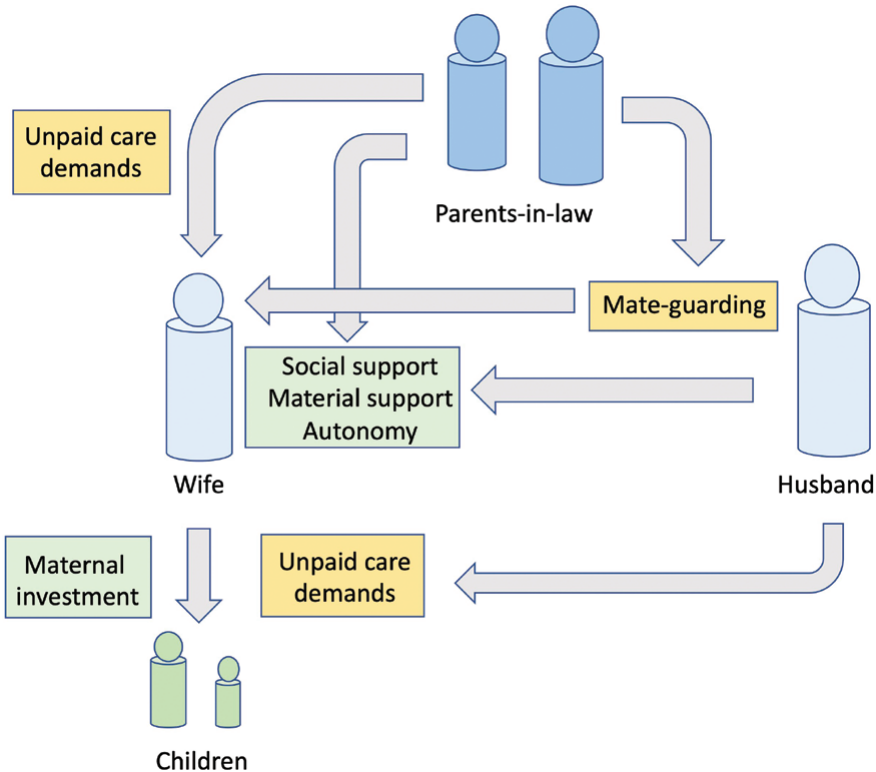
Conceptual framework illustrating potential pathways through which women’s marital household kin (husband and parents-in-law) may generate positive or negative impacts on mental health of the wife/mother. Green boxes indicate positive sources of support, while yellow boxes indicate constraints and demands

**Figure 2** illustrates our evolutionary conceptual approach. We tested competing hypotheses regarding the association of parents-in-law co-residence with maternal mental health and economic autonomy. We further hypothesized that husbands would amplify these effects, on the assumption that common reproductive interests of his kin ultimately underlie family dynamics in patrilineal patrilocal societies.

First, we tested whether maternal outcomes are shaped by conflicts of interest among household members. These hypotheses derive from the expectations that the mother’s husband’s kin may (i) promote household activities that favour grandchildren at a direct cost to maternal health, such as demanding high levels of unpaid care work, and (ii) engage in ‘mate-guarding’ to minimize paternity uncertainty:

H1a. Mothers have better mental health and economic autonomy if parents-in-law are not co-resident

H1b. These benefits are enhanced if husbands are also not co-resident

Second, we tested whether maternal outcomes are shaped by common interests and social support. Following the perspective of Dyble *et al*. [26], co-residency might lead to supportive relationships with her husband’s kin, despite the mother being genetically unrelated to other members. Healthier mothers with greater maternal capital can provide better care for their children, thereby promoting the fitness of her husband’s kin:

H2a. Mothers have worse mental health and economic autonomy if parents-in-law are not co-resident

H2b. These outcomes are worse if the husband is also not co-resident

These hypotheses were proposed before we undertook our statistical analyses.

## METHODS

### Study population

In the Maithili-speaking Madhesi population of our study in rural lowland Nepal, the norm upon marriage is for women to co-reside in extended households with both her parents-in-law and husband. In recent years, men’s extended migration for work has become common in this setting. Strong norms about women’s behaviour prevent women from spending much time outside the house including earning income [51]. Since women are relatively isolated from society, including limited access to their own mother, relations between co-resident members of the household may be especially important for mental health [39].

The women of our study are prone to low income, low education and child undernutrition [52]. Unequal gender relationships in the family underpin these outcomes, with parents-in-law (PIL) occupying the highest status among household members and young mothers the lowest status [53, 54]. Mothers-in-law (MIL) generally supervise their daughters-in-laws’ (DIL) farm work and unpaid care work such as child and elder care, cooking, cleaning and so on [38], which promotes the genetic fitness of family members at the cost of the DIL’s mental health.

The women in our study have few options both in- and outside marriage. Marriage is generally arranged by family members and women are expected to remain married to their spouses. However, men are allowed to remarry, and in some contexts in Nepal (though less common in our study population), take on second wives. This may happen if, for example, their wives do not produce children, especially males, or in the extreme case where a wife may die. In these cases, a woman is generally replaced by another wife, or daughter-in-law. This new daughter-in-law would not only take care of the children from her predecessor, but is also likely to enhance the genetic fitness of her husband and parents-in-law by producing additional children.

### Data collection

We analysed data from the observational Growth Monitoring Study (GMS), conducted in in a subsistence farming population in Dhanusha district in the plains of Nepal. In 2012, 603 children were enrolled at birth and followed up every 28 days until the age of 2 years. Data for this analysis come from the recent follow-up in 2018, and we focus on the 444 mothers who were interviewed.

Research ethics approvals for the GMS were granted by the Nepal Health Research Council for (95/2013, 13/2018) and by the University College London (11345/001). Village Development Committee secretaries consented to the inclusion of clusters. Mothers provided written consent. Trained fieldworkers administered oral questionnaires to mothers.

Mothers were asked about their age (y), ages at marriage (y), caste affiliation, the number of years that they and their husbands had completed in school and who they currently co-resided with.

Maternal mental health of mothers was assessed using the General Health Questionnaire (GHQ-12), a measure of psychosocial distress which is widely used to detect depression and anxiety [49], translated into Maithili. The 12 GHQ questions have been validated for use in Nepal using as a dichotomous score (yes vs no) rather than a Likert scale because of high levels of illiteracy in our study population [44, 55].

Markers of maternal economic autonomy collected by our study included mother’s decision-making power and bargaining power over different aspects of household economic activities. Mothers were asked whether they were the primary decision-makers (yes/no) of major household expenditures and on saving, borrowing or lending money and whether they received and safeguarded household income and did the food shopping. The time per day spent by mothers on household economic activities reflecting their bargaining power included performing farm work (tending crops/animals in their own fields or elsewhere); earning income (from business or money-making activities); doing unpaid care work (domestic chores, caring for children, elderly or sick family members), an important marker of women’s workload [56], which is often unrecognized and unremunerated in formal economic terms [57] or resting or relaxing (leisure activities or sleeping).

Household food security was assessed using the Household Food Insecurity Access Scale [58] as secure, mild, moderate or severe food insecurity. Household wealth was measured using an adapted Demographic Health Survey questionnaire by enumerating household infrastructure and ownership of a range of material goods.

### Data coding

Maternal age was recorded in completed years and summarized as median and interquartile range (IQR). Maternal marriage age was recorded in running years and converted into completed years by subtracting 1. We categorized maternal marriage age as ≤14 years, 15–17 years and ≥18 years to reflect ‘child’, ‘early-adolescent’ and ‘late-adolescent’ marriage [59]. Although the Government of Nepal has recently increased the minimum marriage age from 18 to 20 years [60], few women in our sample married after this age (4% of our cohort). We therefore used the ≥18 years cut-off to define adult marriage, reflecting the universal minimum marriage age established by the United Nations [61].

Education (mothers and their husbands’) was categorized according to the Nepali education system: none, primary (1–5 years) or lower-secondary and above (≥6 years). Caste affiliation included: disadvantaged (Dalit, Muslim), middle (Janajati, other Terai) or advantaged (Yadav, Brahmin, Sudi).

For our primary exposure, we categorized mothers into four groups depending on their co-residence with the following household members: (i) husband (H) only (without co-residence PIL), (ii) neither H nor PIL, (iii) both H and PIL (H+PIL) or (iv) PIL only (where husbands had migrated for work). All households had at least one child aged ~6 years living with the mother.

Our outcome variables comprised markers of low maternal mental health and economic autonomy. Each item in the GHQ-12 was scored 0 (response indicating less anxiety or distress) or 1 (response indicating more anxiety/distress), with a maximum score of 12, and a total score of ≥6 indicating distress [38, 49]. We investigated factors associated with mothers responding negatively to each GHQ item because our interest was in how mothers experienced different aspects of their life and also examined whether they were distressed overall.

The time per day spent by mothers on economic activities reflecting their bargaining power were quantified as lower economic autonomy if they performed >2 h of farm work; did not earn any income; did >5 h of unpaid care work and rested <2 h a day. These categorizations were created based on the distribution of data.

For each outcome related to maternal economic autonomy, we coded the main decision-maker, defined as the person with the decision-making responsibility and person performing the activity in that household as: mother (M), H, M and H jointly (M+H), MIL, or father-in-law (FIL), or MIL and FIL jointly (referred to as PIL). We created binary scores based on median hours spent on each task (**Supplementary Methods**).

Caste affiliation was coded as Disadvantaged (Dalit, Muslim), Mid (Janajati, Other Terai) and Advantaged (Yadav, Brahmin, Sudi). Household Food Security was coded as secure vs any level of food insecurity. A wealth index was constructed using Principal Component Analysis based on household infrastructure and ownership of material goods categorized into quartiles (1 = poorest), as described in **Supplementary Methods**.

### Statistical methods

We follow the widely used epidemiological approach of reporting univariate results first, and second, multivariable logistic models adjusting for a range of key variables. Independent samples *t*-tests (continuous variables) and chi-squared tests (categorical variables) were used to compare (i) women with available vs missing data on PIL co-residence and (ii) maternal and household characteristics stratified by mothers co-residing with H only, neither H nor PIL, both H and PIL, or PIL only.

To test our competing hypotheses, logistic regression models estimated adjusted odds ratios (aOR) with 95% confidence interval (CI) of our exposure variables: co-residence with PIL, H or neither H nor PIL, with maternal mental health and economic autonomy. As explained above, women residing with their natal kin were excluded from our analyses. The reference group was mothers residing with ‘both H+PIL’ because this is generally the norm in this population. We specifically include the co-residence status of the husband as a key exposure rather than a confounding factor because a husband is likely to exert the most influence over their wife’s mental health. For example, studies show that husbands are the most frequent perpetrator of abuse and assault in women in Nepal [62]. We controlled for potential socio-economic confounders, including maternal age, marriage age, maternal/husband’s education, caste, household assets and food security [44].

As recently recommended [63], we report magnitudes of association and their 95% CI but also provide *p*-values in the supplementary tables. Analyses were performed using SPSS 26 (IBM Corp., Armonk, NY) and graphs using Microsoft Excel 365 (Microsoft Corporation, 2021).

## RESULTS

### Sample selection

Data were available on PIL presence in 532 of 603 households: 71 households were not followed-up and therefore had missing data on PIL. The minimal differences between mothers with available vs missing data are unlikely to bias our results (**Supplementary Table S1**). Of 532 households with data on PIL presence, we excluded 39 mothers where their household residence was unclear, 41 mothers who were interviewed in their natal homes and 4 mothers who were in a household other than their marital or natal homes. We excluded a further 4 households due to missing data on maternal and husband’s education, husband’s co-residency presence and who did the food shopping. The composition of our final sample of 444 households is shown in **Supplementary Table S2**. Because of small numbers of household with FIL or MIL only, we combined them into the PIL only or H+PIL categories. All households had at least one child aged ~6 years living with the mother. The final sample distribution was mother living with neither H nor PIL (22%), H only but no PIL (22%), PIL only (31%) or both H+PIL (25%).

### Sample description

Mothers’ median age was 24 years (IQR 7); 29% had married during childhood, 62% during early-adolescence and only 9% at ≥18 years; 72% of mothers and 44% of husbands were uneducated, and 12% and 23% had completed lower-secondary education or above, respectively (**Table 1**). Approximately 36% of households were from the disadvantaged castes and 26% reported any level of food insecurity. Maternal and household characteristics were broadly similar for households with only H and neither PIL nor H, and for households with both H+PIL or only PIL. Women who were not co-residing with PIL were less educated, and their households were poorer and food insecure.

**Table 1.** Summary statistics of sample by parent-in-law and husband co-residence pattern

	Full sample (*n* = 444)	H, no PIL (*n* = 97)	Neither H nor PIL (*n* = 97)	Both H+PIL (*n* = 112)	PIL, no H (*n* = 138)
	Median (IQR)	Median (IQR)	Median (IQR)	Median (IQR)	Median (IQR)
**Mother’s age (y)**	24 (7)	26 (8)	25 (7)	22 (6)	22 (6)
	%	%	%	%	%
**Mother’s marriage age (y)**					
≤14 years	29	34	28	29	25
15–17 years	62	59	64	61	65
≥18 years	9	7	8	10	9
**Mother’s education (y)**					
None	72	79	83	65	67
Primary (1–5 years)	16	13	13	15	19
Lower-secondary and above (≥6)	12	8	4	20	14
**Husband’s education (y)**					
None	44	58	57	31	36
Primary (1–5 years)	33	19	31	37	41
Lower-secondary and above (≥6)	23	23	12	32	23
**Caste** ^a^					
Disadvantaged	36	37	44	29	33
Mid	34	34	30	29	41
Advantaged	30	29	26	42	26
**Household assets**					
1: Poorest	26	41	28	19	18
2	24	22	29	20	26
3	25	21	26	26	28
4: Richest	25	16	17	35	28
**Household food insecurity**	26	39	28	22	18

IQR, interquartile range. %, percentage. Husband (H), Parents-in-law (PIL).

^a^Caste groups include Disadvantaged (Dalit, Muslim), Mid (Janajati, Other Terai) and Advantaged (Yadav, Brahmin, Sudi). The group of women who were living with neither relative does not include women were residing with their natal kin.

### Unadjusted results

**Table 2** summarizes maternal mental health outcomes according to co-residence patterns. Mothers were less likely to lose sleep, feel unhappy, depressed or worthless if they lived with both H+PIL or only PIL, compared to living with only H or neither relative. However, mothers living with PIL, with or without H, were also more likely to feel incapable of making decisions and constantly under strain. Women co-residing with only H were more likely to feel they were not playing a useful part in life, unconfident, worthless and be distressed overall.

**Table 2.** Maternal psychological distress by parent-in-law and husband co-residence pattern

	Full sample (*n*=444)	H, no PIL (*n*=97)	Neither H nor PIL (*n*=97)	Both H+PIL (*n*=112)	PIL, no H (*n*=138)
	%^a^	%	%	%	%
Unable to concentrate	21	19	19	23	23
Lost sleep over worry	47	57	57	38	40
Not playing useful part	55	67	55	47	53
Incapable of making decisions	48	42	47	51	50
Constantly under strain	17	12	14	21	19
Can’t overcome difficulties	20	21	20	18	20
Unable to enjoy daily activities	4	4	4	2	5
Unable to face problems	31	31	33	26	34
Unhappy and depressed	38	46	43	31	33
Losing self-confidence	41	50	42	41	33
Feeling worthless	42	65	45	33	31
Overall unhappy	4	6	7	0	4
**Overall distressed (score ≥6)**	22	31	23	20	15

H, Husband; PIL, Parents-in-law.

^a^Frequencies of mothers who answered ‘yes’ to binary questions on whether they experienced these problems. The group of women who were living with neither relative does not include women were residing with their natal kin.

**Figure 3.**
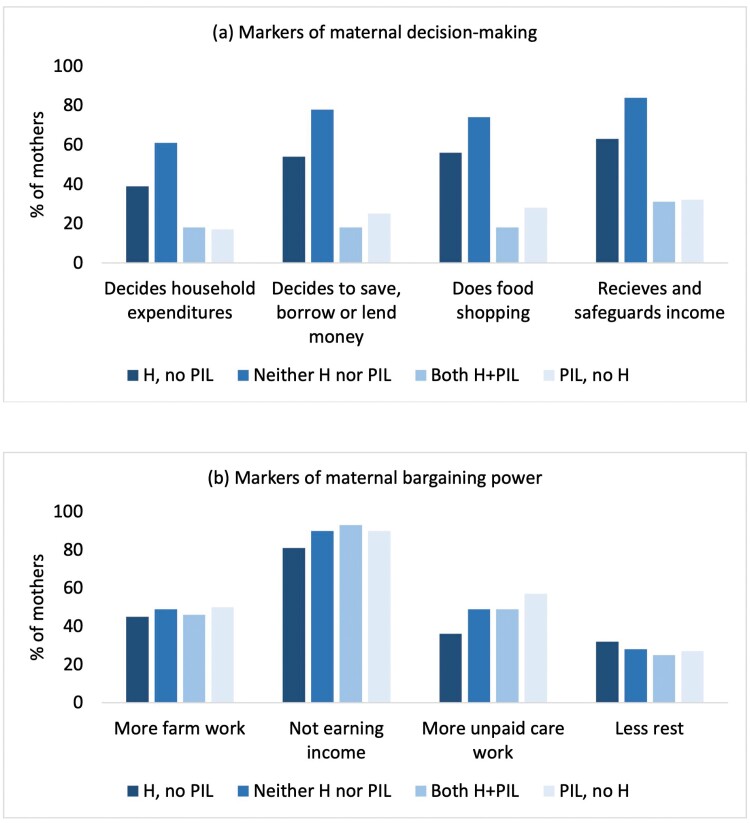
Markers of maternal economic autonomy by parent-in-law and husband co-residence pattern. **Figure 3a** illustrates the proportion of mothers with greater economic autonomy in household economic decision-making. It shows the proportion of mothers who (a) answered ‘yes’ to whether they made decisions about household expenditures, and on saving, borrowing or lending money, did the food shopping, and received and safeguard household income, stratified by co-residence of Husband (H) only but no Parents-in-law (PIL), neither H nor PIL, both H+PIL, or PIL but no H. **Figure 3b** illustrates the proportion of mothers with lower economic autonomy reflecting their bargaining power. It shows the proportion of women who reported spending more time on farm work, not earning income, performing more unpaid care work, or resting less, by co-residence patterns. Data are given in **Supplementary Table S3**.

**Figure 3** presents crude frequencies of our markers of maternal economic autonomy by co-residence pattern (data in **Supplementary Table S3**). The proportion of mothers involved in four aspects of household economics was lower if they lived with PIL only, or both H+PIL, compared to those who lived with their husband only or with neither H nor PIL (**Fig. 3a**). There were no differences in the proportion of mothers who reported spending more time on farm work or resting less by co-residence pattern (**Fig. 3b**). However, a lower proportion of mothers co-residing with their H only reported not earning income or performing more unpaid care work, compared with those co-resident with either PIL, both H+PIL or neither relative.

**Figure 4.**
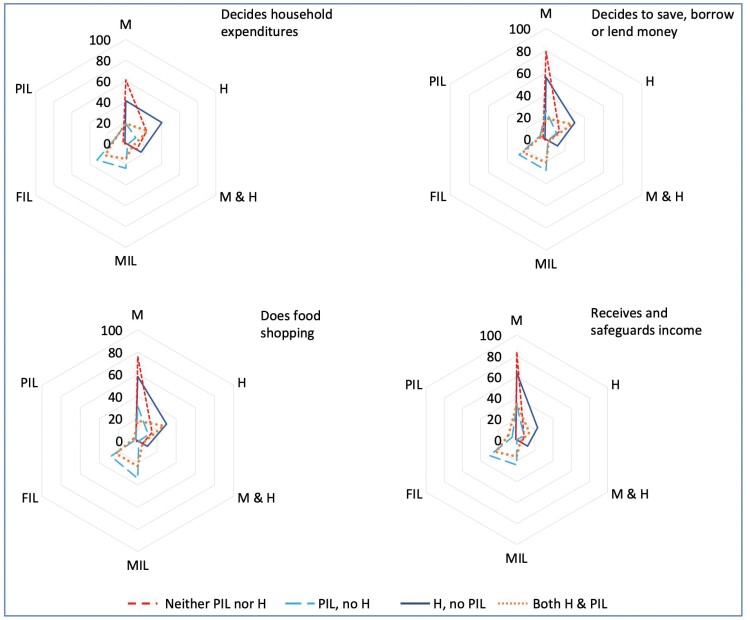
Household member involvement in household economics by co-residence pattern. **Figure 4** illustrates the frequency of the household member reported by the mother to be the main decision-maker on household expenditures and on saving, borrowing, or lending money, and the main person who did the food shopping, and received and safeguard household income, stratified by the four types of households. The relatives who made decisions included: Mother (M), Husband (H), M and H together, Mother-in-law (MIL), Father-in-law (FIL) or Parents-in-law together (PIL). Results are stratified by co-residence of neither H nor PIL; H, but no PIL; PIL, but no H; both H+PIL. The data clearly show the effect of co-resident and not co-resident PILs on the mother’s decision-making and involvement in household economic activities. Data are given in **Supplementary Table S4**

**Figure 4** shows the frequency of the household member reported by the mother to be the main decision-maker or person performing tasks related to household economics, stratified by the four types of households (data in **Supplementary Table S4**). In households with co-resident PIL, either the MIL or FIL was most frequently the usual decision-maker, irrespective of H co-residence. In households where PIL only were co-resident, the MIL and M made decisions on food shopping, whereas in household with both H+PIL, the PIL and H made these decisions. In households with no co-resident PIL, the mother herself was most frequently the usual decision-maker for household expenditures and food shopping, regardless of H co-residence. However, in some cases, the H was the main decision-maker even if not resident, indicating communicated decisions.

### Adjusted logistic regression models testing competing hypotheses

**Figure 5.**
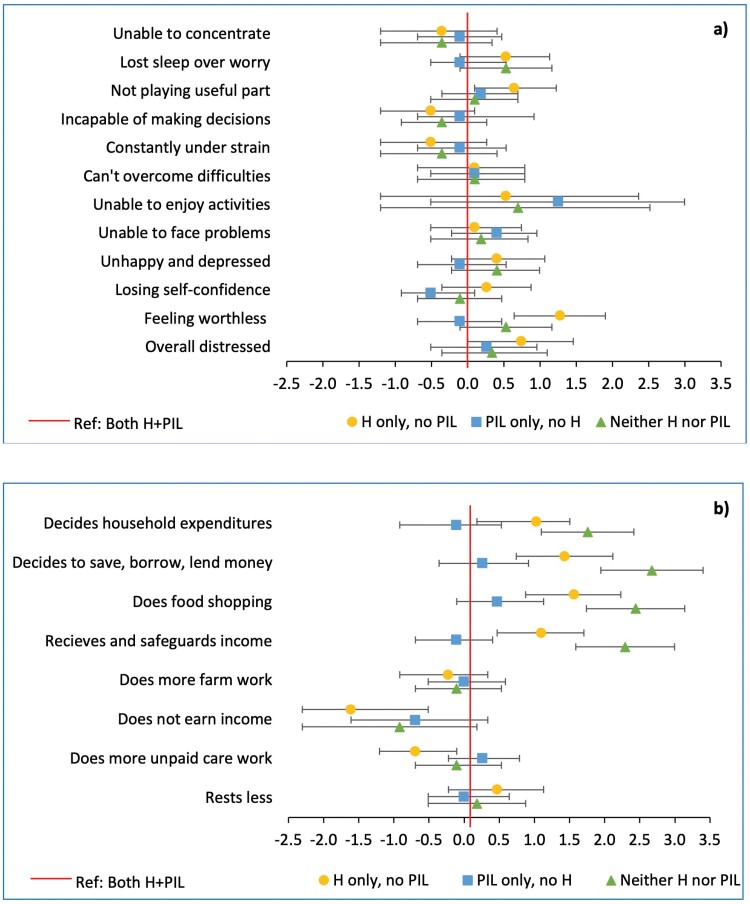
Maternal (a) mental health and (b) economic autonomy by co-residence pattern. **Figure 5** illustrates adjusted Log Odds Ratios for the mother reporting that (a) that she had adverse mental health outcomes and (b) that she had decision-making power and bargaining power over household economic activities. The exposures were co-residing with Husband (H) only, with Parents-in-law (PIL) only, or neither H nor PIL. The reference group was co-residing with both H+PIL. Large 95% Confidence Intervals for mothers reporting they were unable to enjoy daily activities were due to very few women answering yes to this question. Models adjusted for: maternal age, maternal marriage age, maternal/husbands’ education, caste, household assets and food security. Data are given in **Supplementary Tables S5 and S6**

**Figure 5a** shows log aOR for these associations, adjusting for maternal age, marriage age, maternal/husband’s education, caste, household assets and food security (data in **Supplementary Table S5**). Regarding mental health, compared to mothers residing with both H+PIL, co-residence with husband only or neither PIL nor H, was associated with higher odds of mothers losing sleep, feeling unhappy or depressed and feeling worthless. Co-residence with husbands only was associated with higher odds of mothers feeling they were not playing a useful part in life, low self-confidence and overall distress but also with being more capable of making decisions. Relative to living with both H+PIL, women living with PIL only had similar mental health, which suggests that presence of the H did not impact women’s distress in households where PIL were co-resident.

Regarding economic autonomy, **Fig. 5b** shows that relative to living with both H+PIL, co-residence with H only or neither H nor PIL was associated with increased odds of mothers being the main decision-maker and their involvement in all aspects of household economics (data in **Supplementary Table S6**). Relative to living with both H+PIL, co-residence with PIL only was not associated with any difference in maternal economic autonomy.

## DISCUSSION

Our study was conducted in a Maithili-speaking Madhesi society, where women are socially isolated, largely restricted to the husband’s home, and tend to be accompanied by a relative when venturing outside the home. From an evolutionary perspective, this isolation may be considered beneficial for the fitness of the husband and his genetic kin, both through maximizing the wife’s childcare and unpaid care work (reproductive investment), while also minimizing her opportunities for extra-pair mating. However, as recognized by Dyble *et al*. [26], supporting the mother could also bring fitness benefits to marital kin, through promoting maternal capital and increasing the survival and health of the offspring, and subsequently the quantity and phenotypic quality of grandchildren. These contrasting strategies are expected to impact both of the outcomes we investigated, maternal mental health and economic autonomy. Our study therefore explored how these outcomes varied by household composition.

We tested competing hypotheses, to identify whether specific types of relative were more likely to provide support or constraint to mothers. By using the co-residence of both husband and parents-in-law as the reference group, we could evaluate maternal outcomes when specific categories of relative were not co-resident, and thereby learn about the influence of these relatives on maternal outcomes. We found evidence for both of our overarching hypotheses: individuals who are genetically unrelated to mothers may provide both support and constraint, depending on the outcome and the category of relative.

When parents-in-law but not husbands were co-resident, mothers had similar mental health outcomes as the reference group. Conversely, when the husband was present but not the parents-in-law, maternal mental health was worse, with mothers reporting losing sleep, feeling unhappy and worthless, and greater overall distress. When neither category of relative was present, maternal outcomes did not differ from the reference group. Overall, this suggests that parents-in-law provided some support to mothers, thereby improving their mental health. Importantly, the fact that the contrast in the distress score was greatest between both parents-in-law and husband being co-resident (lowest score), versus husband only (highest), suggests that parents-in-law may actively have reduced negative impacts of the husband. These differences are of public health significance, as mothers were two times more likely to be distressed if they co-resided with only their husbands, compared to both husband and parents-in-law.

When parents-in-law but not husbands were co-resident, mothers had similarly low economic autonomy as those in the reference group. **Figure 4** shows that when they were co-resident, parents-in-law were the primary economic decision-makers in the household, regardless of whether the husband was co-resident or not. Conversely, when the husband was present but not the parents-in-law, maternal autonomy increased, and paradoxically, this increment was further enhanced if the husband himself was not co-resident, having migrated for work. In the absence of parents-in-law, the mother was the primary economic decision-maker in the household. These patterns suggest that overall, parents-in-law inhibited maternal economic autonomy, whereas husbands promoted it. This can be attributed to women living without their parents-in-law being required to take more responsibility for the household, though husbands often continued to make economic decisions if they were overseas.

We interpret these two sets of results in combination as suggesting that mothers are exposed to trade-offs: different categories of marital kin balance the control and constraint of women with different forms of support. We suggest that wives in this population may be overall subjected to two forms of mate-guarding, that differ in how they relate to the household’s need for women to express economic autonomy. When co-resident, parents-in-law appear to constrain women’s autonomy, but having negated their agency, a form of mate-guarding, they also seem to provide some social support that benefitted their mental health. In contrast, husbands appear to promote the economic autonomy of their wife, and reduce her unpaid care work, but by undermining her sense of worth and mental health, husbands may also operationalize an alternative tactic for mate-guarding. We found some support for this second hypothesis, as when parents-in-law were not co-resident, mental health outcomes were better for women whose husband was also not co-resident, compared with those where he was. As the majority of mothers in our sample were either completely uneducated or had only primary levels of education, the co-residence status of PILs and H, rather than her education, are likely to be the key factors associated with maternal mental health and economic autonomy.

In this study, we focused on continuous markers of mental health and autonomy that can be measured across the whole population. However, other work has focused on more extreme markers of the costs inflicted to women, such as violence by one or both of husband and parents-in-law [34, 35, 64, 65]. Women in this society are therefore likely to be familiar with the threat of violence, reducing their incentive to resist familial constraints on their autonomy.

Our findings are consistent with other studies noting mental health benefits of social support [39, 44]. Regarding autonomy, other studies have also found parents-in-law to be the most frequent household economic decision-makers [19] but also that non-resident husbands may continue to make decisions [39]. Other studies have also found that parents-in-law reduce mothers’ income earning opportunities [44], which is of interest given our prediction that parents-in-law would act in the reproductive interests of their son.

Our measure of unpaid care work included support for elder members of the household. This direction of support is also given emphasis through the lens of the patriarchal bargain framework [36], which considers that the interests of maintaining senior household member’s health, autonomy and food security shape the experience of younger members. However, Gram *et al*. also found evidence of husbands pushing back against parents-in-law influence, by amassing enough resources to eventually form a nuclear household with their wives [53]. This may relate both to the husband’s own reproductive interests, but also to his interest in maintaining a good quality relationship (pair-bonding) with his wife [66].

In a society where decisions are typically made collectively, and where the opinion of older family members reflects their greater experience and authority, women lacking co-resident family members may find the pressure to run the household and taking on decision-making responsibility stressful, as reported in other studies [67]. Consequently, a lack of economic autonomy may not be perceived by some mothers as a sign of disempowerment, as they may not want the responsibility of making decisions, which in itself may cause distress, especially in the absence of their husbands [68]. Efforts to avoid stressful situations may be a covert form of agency or empowerment in this population [50]. While acknowledging the diversity of definitions of empowerment [50], we use it as a broader concept reflecting women’s ability to overcome barriers and achieve their goals and desired outcomes.

In the context of our study, households are primarily patrilocal and patriarchal. Future studies could test our competing evolutionary hypotheses more broadly, by comparing patrilocally and matrilocally married women, with and without husbands and either parents-in-law or parents. For example, in matrilineal societies, a mother’s own mother may help her to meet high costs of reproductive investment, if demanded by her husband. Whether families are characterized by cooperation or conflict may also be elucidated by considering factors such as maternal age, income earning opportunities outside the home, parity and family members beyond parents-in-law, parents and husbands, all of which may shape maternal mental health and economic autonomy. For example, our recent fieldwork in this cohort will explore the implications for mental health of women becoming increasingly related to other group members as they age, by transitioning to the role of mother-in-law [69].

### Strengths and limitations

Among the strengths of our study was the large sample size in a particularly vulnerable population, with data on several markers of maternal mental health and economic autonomy, including involvement in household economic decision-making and activities. Our study design allowed us to analyse how different markers of maternal psychological distress (each of the 12 GHQ questions) and economic autonomy (both decision-making power and bargaining power) were associated independently with the co-residence of key family members.

Limitations included a lack of data on parents-in-law and husband mental health and economic autonomy. By excluding a small number of mothers who were interviewed in their natal household, we may have lost those whose mental health outcomes were most adversely affected by their marital kin. However, we did not know whether these women had left their marital home altogether, or under what circumstances they were staying there.

We also lacked some relevant variables. Due to missing values, we did not include maternal age at first pregnancy in our analyses, or factors underlying variability in work demands such as land ownership. Maternal parity was only measured at baseline and therefore excluded from analyses. Assessing how co-residence and economic contributions of other household members beyond parents-in-law and husbands may have impacted maternal mental health and economic autonomy was beyond the scope of this paper. Our marker of maternal economic bargaining power is an imperfect measure as the monetary size of earnings (upon which we have no data) is likely to matter more than time spent working for pay. We do not have data specifically on mate-guarding, and interpret the restricted physical mobility of women, their lack of economic autonomy and their psychological stress as markers of this strategy.

Finally, although the women in our study are similar to other South Asian women in their restricted physical mobility, the associations we found are relevant to patrilocal family arrangements, but may not generalize to other populations, including matrilineal and polygamous societies.

## CONCLUSIONS

Overall, our results indicate that mothers face trade-offs regarding their household activities, and may experience both mental health benefits and costs through co-residence with other family members, alongside variable levels of economic autonomy [26]. As recently highlighted [26], a person related by marriage (affine) may gain fitness benefits by providing support to an unrelated individual, who will carry their genes via reproduction with a relative of the affine. Both husbands and parents-in-law could gain such fitness pay-offs, by supporting mothers and reducing conflict and distress, with positive effects on the quality of her offspring [24]. However, marital kin could also increase their fitness by reducing paternity uncertainty, and we suggest that this strategy is evident in our study population, as indicated by very restricted physical mobility and social contact outside the family.

Our interest here is in maternal mental health in its own right, irrespective of its implications for Darwinian fitness. Our findings suggest that parents-in-law offer women greater support than do their husbands, which may be possible because they also restrict their economic autonomy. When parent-sin-law are not co-resident, husbands may use more controlling tactics (psychosocial pressure, potentially leading to distress), to counterbalance their requirement for greater economic autonomy by their wives. Future work could consider the mental health of the parents-in-law and husbands from the same perspective. Overall, we favour a nuanced framework that acknowledges negotiation and nurturing among genetically unrelated family members, as well as hierarchical power dynamics.

## Supplementary Material

eoad020_suppl_Supplementary_Material

## Data Availability

Requests to access the dataset for replicating the analyses, through a data sharing agreement, should be directed to Dr Naomi Saville, n.saville@ucl.ac.uk.
