# Supplementary material for: Maternal mental health and economic autonomy in lowland rural Nepal: Do parents-in-law provide constraint or support?
Source: Evol Med Public Health. 2023 Jul 4;11(1):229–43. doi: 10.1093/emph/eoad020 (PMC10355796; doi:10.1093/emph/eoad020)
Supplement: eoad020_suppl_Supplementary_Material [file eoad020_suppl_supplementary_material.docx]

**Maternal mental health and economic autonomy in lowland rural Nepal: do parents-in-law provide constraint or support?**

**Supplementary materials**

**Supplementary methods**

**Description of the Principal Component Analysis of wealth**

The first principal component was taken as a marker of wealth because it explained 35.6% of the variance, compared to 13.2% and 10.5% respectively for the second and third components. This indicates a strong correlation between the first PCA component and the variables related to wealth. The nine variables comprising the first principal component included (factor loading): materials used for walls (0.426), flooring (0.412) and roofing (0.389); number of rooms used for sleeping (0.341), toilet type (0.335), ownership of motorbike (0.318) and colour television (0.268), access to electricity (0.223), and water source (0.215).

**Description of binary scores of maternal economic bargaining power**

Binary scores of median hours spent on each task by mothers were defined as follows: spending more time on farming (>1 hour/day either on their own or others’ farm) and unpaid care work (>4 hours/day), not earning income, and having less opportunity to rest/relax (<2 hours/day).

**Table S1. Differences between households with no data vs data on parents-in-law co-residence**

|  | **No data on PIL co-residence, lost to follow-up** (*n*=71) | | **Data on PIL co-residence**  (*n*=532) | | **Difference** |
| --- | --- | --- | --- | --- | --- |
|  | **Median** | **IQR** | **Median** | **IQR** | ***p*-value^1^** |
| **Maternal age (y)** | 23 | 6 | 24 | 7 | 0.591 |
| **Maternal marriage age (y)** | 16 | 1 | 16 | 2 | 0.262 |
|  | **F** | **%** | **F** | **%** | ***p*-value^2^** |
| **Maternal education**^3^ |  |  |  |  | 0.139 |
| None | 43 | 61 | 379 | 71 |  |
| Primary to lower secondary | 15 | 21 | 89 | 17 |  |
| Secondary above | 13 | 18 | 62 | 12 |  |
| **Husband’s education**^4^ |  |  |  |  | 0.023 |
| None | 20 | 28 | 237 | 45 |  |
| Primary to lower secondary | 27 | 38 | 171 | 32 |  |
| Secondary above | 24 | 34 | 123 | 23 |  |
| **Caste**^4,5^ |  |  |  |  | 0.017 |
| Disadvantaged | 14 | 20 | 186 | 35 |  |
| Mid | 35 | 49 | 184 | 35 |  |
| Advantaged | 22 | 31 | 161 | 30 |  |

*n*, number. IQR, interquartile range. F, Frequency. %, percentage. Parents-in-law (PIL). ^1^Krustal-Wallis test. ^2^Chi-squared test. ^3^Data on PIL presence *n*=530. ^4^Data on PIL presence *n*=531. ^5^Caste groups include Disadvantaged (Dalit, Muslim), Mid (Janajati, Other Terai) and Advantaged (Yadav, Brahmin, Sudi).

**Table S2. Composition of 444 households in sample**

| **Household member co-residence presence^#^** | **Percentage in each category** | **Percentage (rounded off) after grouping into four main categories** |
| --- | --- | --- |
| **Neither MIL, FIL nor H** | 21.8 | 22 |
| **H only, no MIL or FIL** | 21.8 | 22 |
| **PIL only, MIL and/or FIL, no H** | 21.7 | 31 |
| **Both H+PIL (MIL and/or FIL)** | 15.5 | 25 |
| MIL and H, no FIL (combined with both) | 6.1 | NA |
| FIL and H, no MIL (combined with both) | 3.6 |  |
| MIL only, no FIL, no H (combined with PIL only) | 5.9 |  |
| FIL only, no MIL, no H (combined with PIL only) | 3.6 |  |
| Total | 100 | 100 |

Mother-in-law (MIL), Father-in-law (FIL), Husband (H), Parents-in-law (PIL). NA, not applicable. Cells shaded in green and blue were combined respectively into four main categories, shown in bold in the first column. The rounded off percentage for these four main household categories are shown in the third column. The ‘neither’ category does not include women who were residing with their natal kin at the time of the study. ^#^All households had at least one child aged ~6 years living with the mother.

**Table S3. Data for Figures 3a and 3b: Markers of maternal economic autonomy by co-residence pattern**

|  |  | **Household member with whom the mother co-resides** | | | |  |
| --- | --- | --- | --- | --- | --- | --- |
|  | **Full sample** (*n*=444) | **H, no PIL** (*n*=97) | **Neither H nor PIL**  (*n*=97) | **Both H+PIL**  (*n*=112) | **PIL, no H**  (*n*=138) | **Difference** |
|  | **%** | **%** | **%** | **%** | **%** | ***p*-value^3^** |
| Figure 3a: markers of maternal economic decision-making power | | | | | | |
| Decisions on household expenditure | 32 | 39 | 61 | 18 | 17 | <0.001 |
| Decisions on saving, borrowing or lending money | 41 | 54 | 78 | 18 | 25 | <0.001 |
| Does food shopping | 42 | 56 | 74 | 18 | 28 | <0.001 |
| Receives and safeguards household income | 50 | 63 | 84 | 31 | 32 | <0.001 |
| Figure 3b: markers of maternal bargaining power | | | | | | |
| ore farm work (>2 hr/day) | 48 | 45 | 49 | 46 | 50 | 0.876 |
| Not earning income | 89 | 81 | 90 | 93 | 90 | 0.062 |
| More unpaid care work (>5 hrs/day) | 49 | 36 | 49 | 49 | 57 | 0.017 |
| Less rest (<2 hrs/day) | 28 | 32 | 28 | 25 | 27 | 0.719 |

*n*, number. *%*, percentage. Husband (H), Parents-in-law (PIL). ^3^Chi-squared test. The ‘neither’ category does not include women who were residing with their natal kin at the time of the study.

**Table S4. Data for Figure 4: Household member involvement in economic decision-making by co-residence pattern**  (*n*=414)^1^

|  | **Decision-maker** | | | | | |  |
| --- | --- | --- | --- | --- | --- | --- | --- |
|  | **M** | **H** | **Both M + H** | **MIL** | **FIL** | **PIL** | **Total** |
| **Household type, by co-residence** | **%** | **%** | **%** | **%** | **%** | **%** | **%** |
| **Decides on household expenditures** | | | | | | |  |
| H, but no PIL | 41 | 40 | 17 | 0 | 1 | 1 | 100 |
| Neither H nor PIL | 61 | 23 | 12 | 0 | 1 | 3 | 100 |
| PIL, but no H | 19 | 11 | 2 | 24 | 32 | 11 | 100 |
| Both H+PIL | 19 | 24 | 7 | 15 | 23 | 11 | 100 |
| **Decides on saving, borrowing or lending money** | | | | | | |  |
| H, but no PIL | 56 | 30 | 12 | 0 | 1 | 1 | 100 |
| Neither H nor PIL | 79 | 14 | 3 | 0 | 1 | 3 | 100 |
| PIL, but no H | 26 | 11 | 2 | 28 | 28 | 6 | 100 |
| Both H+PIL | 19 | 27 | 3 | 21 | 24 | 5 | 100 |
| **Does the food shopping** | | | | | | |  |
| H, but no PIL | 58 | 30 | 10 | 0 | 0 | 2 | 100 |
| Neither H nor PIL | 76 | 15 | 5 | 0 | 2 | 2 | 100 |
| PIL, but no H | 31 | 11 | 1 | 34 | 28 | 2 | 100 |
| Both H+PIL | 18 | 27 | 5 | 23 | 23 | 4 | 100 |
| **Receives and safeguards household income** | | | | | | |  |
| H, but no PIL | 64 | 23 | 12 | 0 | 0 | 1 | 100 |
| Neither H nor PIL | 84 | 8 | 6 | 0 | 0 | 1 | 100 |
| PIL, but no H | 33 | 8 | 0 | 24 | 30 | 5 | 100 |
| Both H+PIL | 34 | 14 | 4 | 16 | 23 | 9 | 100 |

*n*, number. *%*, percentage. The table reports the frequency of each household member reported by the mother to be the main decision-maker over categories of economic decision making or activity, stratified by the four types of households. The relatives who made decisions included: Mother (M), Husband (H), M and H together, Mother-in-law (MIL), Father-in-law (FIL) or both MIL and FIL together (parents-in-law, PIL). The ‘neither’ category does not include women who were residing with their natal kin at the time of the study.

**Table S5. Data for Figure 5a: Associations of maternal mental health with co-residence pattern**

|  | **Reference group** | **Exposures** | | | | | | | | |
| --- | --- | --- | --- | --- | --- | --- | --- | --- | --- | --- |
| **Outcome variables** | **Both H+PIL** | **H, no PIL** | | | **Neither PIL nor H** | | | **PIL, no H** | | |
|  | **aOR** | **aOR** | **95%CI** | ***p*-value** | **aOR** | **95%CI** | ***p*-value** | **aOR** | **95%CI** | ***p*-value** |
| Unable to concentrate | 1.0 | 0.7 | 0.3, 1.5 | 0.392 | 0.7 | 0.3, 1.4 | 0.354 | 0.9 | 0.5, 1.6 | 0.681 |
| Lost sleep over worry | 1.0 | 1.7 | 0.9, 3.1 | 0.079 | 1.7 | 0.9, 3.2 | 0.063 | 0.9 | 0.6, 1.7 | 0.989 |
| Not playing useful part | 1.0 | 1.9 | 1.1, 3.4 | 0.031 | 1.1 | 0.6, 2.0 | 0.655 | 1.2 | 0.7, 2.0 | 0.490 |
| Incapable of making decisions | 1.0 | 0.6 | 0.3, 1.1 | 0.085 | 0.7 | 0.4, 1.3 | 0.259 | 0.9 | 0.5, 2.5 | 0.712 |
| Constantly under strain | 1.0 | 0.6 | 0.3, 1.3 | 0.210 | 0.7 | 0.3, 1.5 | 0.380 | 0.9 | 0.5, 1.7 | 0.752 |
| Can’t overcome difficulties | 1.0 | 1.1 | 0.5, 2.2 | 0.840 | 1.1 | 0.5, 2.2 | 0.845 | 1.1 | 0.6, 2.2 | 0.700 |
| Unable to enjoy daily activities | 1.0 | 1.7 | 0.3, 10.6 | 0.567 | 2.0 | 0.3. 12.4 | 0.456 | 3.5 | 0.6, 20.0 | 0.157 |
| Unable to face problems | 1.0 | 1.1 | 0.6, 2.1 | 0.715 | 1.2 | 0.6, 2.3 | 0.523 | 1.5 | 0.8, 2.6 | 0.163 |
| Unhappy and depressed | 1.0 | 1.5 | 0.8, 2.9 | 0.156 | 1.5 | 0.8, 2.7 | 0.194 | 0.9 | 0.5, 1.7 | 0.925 |
| Losing self-confidence | 1.0 | 1.3 | 0.7, 2.4 | 0.347 | 0.9 | 0.5, 1.6 | 0.722 | 0.6 | 0.4, 1.1 | 0.114 |
| Feeling worthless | 1.0 | 3.6 | 1.9, 6.7 | <0.001 | 1.7 | 0.9, 3.2 | 0.075 | 0.9 | 0.5, 1.6 | 0.747 |
| Overall distressed (score of ≥6) | 1.0 | 2.1 | 1.0, 4.3 | 0.042 | 1.4 | 0.7, 3.0 | 0.320 | 1.3 | 0.6, 2.6 | 0.438 |

aOR, adjusted odds ratio. CI, Confidence Interval. Each row reports results from separate multivariable logistic regression models. The left-most column indicates the outcome/dependent variable, which are markers of adverse maternal mental health outcomes, to which mothers answered ‘yes’. The second column from the left indicates the reference group: both Husband (H) + Parents-in-law (PIL). The third column indicates the three exposures, which were mothers co-resident with H only but no PIL, neither PIL nor H, or PIL only but no H. Models adjust for: maternal age, maternal marriage age, maternal/husbands’ education, caste, household assets and food security. The ‘neither’ category does not include women who were residing with their natal kin at the time of the study.

**Table S6. Data for Figure 5b: Markers of maternal economic autonomy**

|  | **Reference group** | **Exposures** | | | | | | | | |
| --- | --- | --- | --- | --- | --- | --- | --- | --- | --- | --- |
| **Outcome variables** | **Both H+PIL** | **H, no PIL** | | | **Neither PIL nor H** | | | **PIL, no H** | | |
|  | **aOR** | **aOR** | **95%CI** | ***p*-value** | **aOR** | **95%CI** | ***p*-value** | **aOR** | **95%CI** | ***p*-value** |
| Maternal economic decision-making power | | | | | | | | | | |
| Decides on household expenditure | 1.0 | 2.8 | 1.2, 4.5 | 0.015 | 5.8 | 3.0, 11.2 | <0.001 | 0.9 | 0.4, 1.7 | 0.719 |
| Decides on saving, borrowing or lending money | 1.0 | 4.2 | 2.1, 8.3 | <0.001 | 14.5 | 7.0, 30.0 | <0.001 | 1.3 | 0.7, 2.5 | 0.425 |
| Does food shopping | 1.0 | 4.8 | 2.4, 9.3 | <0.001 | 11.5 | 5.7, 23.1 | <0.001 | 1.6 | 0.9, 3.1 | 0.135 |
| Receives and safeguards income | 1.0 | 3.0 | 1.6, 5.5 | <0.001 | 9.9 | 4.9, 20.0 | <0.001 | 0.9 | 0.5, 1.5 | 0.668 |
| Maternal economic bargaining power | | | | | | | | | | |
| Does more farm work (>2 hr/day) | 1.0 | 0.8 | 0.4, 1.4 | 0.477 | 0.9 | 0.5, 1.7 | 0.849 | 1.0 | 0.6, 1.8 | 0.880 |
| Does not earn income | 1.0 | 0.2 | 0.1, 0.6 | 0.004 | 0.4 | 0.1, 1.2 | 0.094 | 0.5 | 0.2, 1.4 | 0.221 |
| Does more unpaid care work (>5 hrs/day) | 1.0 | 0.5 | 0.3, 0.9 | 0.040 | 0.9 | 0.5, 1.7 | 0.828 | 1.3 | 0.8, 2.2 | 0.334 |
| Rests less (<2 hrs/day) | 1.0 | 1.6 | 0.8, 3.1 | 0.152 | 1.2 | 0.6, 2.4 | 0.536 | 1.0 | 0.6, 1.9 | 0.907 |

aOR, adjusted odds ratio. CI, Confidence Interval. Each row reports results from separate multivariable logistic regression models. The left-most column indicates the outcome/dependent variable, which are markers of maternal economic decision-making power and economic bargaining power. The second column from the left indicates the reference group: both Husband (H) + Parents-in-law (PIL). The third column indicates the three exposures, which were mothers co-resident with H only but no PIL, neither PIL nor H, or PIL only but no H. Models adjust for: maternal age, maternal marriage age, maternal/husbands’ education, caste, household assets and food security. The ‘neither’ category does not include women who were residing with their natal kin at the time of the study.
